# Single-cell multi-omics in biliary tract cancers: decoding heterogeneity, microenvironment, and treatment strategies

**DOI:** 10.1186/s43556-025-00330-2

**Published:** 2025-10-15

**Authors:** Nannan Tang, Jiatong Li, Ao Gu, Mengyao Li, Yingbin Liu

**Affiliations:** 1 State Key Laboratory of Systems Medicine for Cancer, Shanghai Cancer Institute, Renji Hospital, Shanghai Jiao Tong University School of Medicine, Shanghai, China; 2 Department of Biliary-Pancreatic Surgery, Renji Hospital, Shanghai Jiao Tong University School of Medicine, Shanghai, China

**Keywords:** Biliary tract cancers, Single-cell multi-omics, Tumor microenvironment, Tumor heterogeneity, Personalized treatment

## Abstract

Biliary tract cancer (BTC) is a highly heterogeneous and aggressive gastrointestinal malignancy, marked by a high mortality rate and limited treatment efficacy. The primary contributing factors include the absence of reliable early detection methods, the anatomical intricacy of the biliary system, the inherently aggressive tumor biology, and the restricted effectiveness of systemic therapies. A profound understanding of molecular characteristics and clinically relevant emerging biomarkers is essential for advancing BTC treatment strategies. Recent developments in single-cell multi-omics technologies have enabled the analysis of genetic, transcriptomic, proteomic, and metabolomic data at the single-cell resolution, thereby uncovering the heterogeneity and complexity of tumor biology. These techniques provide critical insights into the diversity of immune cell populations within the tumor microenvironment (TME) and offer novel perspectives on tumor progression and potential therapeutic interventions. While single-cell technologies have significantly advanced the study of solid tumors, their application in BTC remains nascent, with a paucity of comprehensive reviews. This review systematically integrates single-cell genomics, transcriptomics, and epigenomics data to construct a cross-omics molecular atlas of BTC. It highlights the utility of single-cell multi-omics technologies in elucidating tumor heterogeneity, microenvironment remodeling, and clonal evolution in biliary tumors, while thoroughly analyzing their implications for clinical outcomes. Furthermore, this review explores personalized treatment strategies informed by single-cell technologies and underscores the significance of these technologies as indispensable tools for unraveling the complexity of BTC and fostering mechanism-based therapeutic innovation.

## Introduction

Biliary tract cancers (BTCs), including cholangiocarcinoma (CCA), gallbladder cancer (GBC), and ampulla of vater cancer (AVC), are rare yet highly heterogeneous and aggressive malignancies of the gastrointestinal tract that arise from the bile duct and gallbladder epithelium [1]. CCA is further classified based on its anatomical location into intrahepatic cholangiocarcinoma (iCCA) and extrahepatic cholangiocarcinoma (eCCA), with eCCA subdivided into distal cholangiocarcinoma (dCCA) and perihilar cholangiocarcinoma (pCCA) [2–4]. Global epidemiological data indicate that BTCs represent roughly 3% of all digestive system malignancies. However, surgical resection is the preferred and currently the only potentially curative treatment for BTCs, the majority of patients present with advanced-stage disease, leaving only about 20% eligible for surgery [5]. Even following R0 resection, the recurrence rate remains high, reaching up to 67% within the first year post-surgery [6, 7]. Despite advances in oncology, BTCs remain challenging to treat due to late diagnosis, limited therapeutic options, and intrinsic resistance to conventional therapies. The 5-year survival rate for BTCs patients is dismal, underscoring the urgent need for a deeper understanding of their biological complexity to develop effective treatments.

The clinical profile of BTCs characterized by high mortality and poor treatment response underscores the underlying complexity of the disease, including factors such as tumor microenvironment (TME) remodeling, epigenetic regulation, and clonal evolution [8–10]. The TME, characterized by the dynamic interactions between cancer cells and their surrounding environment, has been identified as a key factor in tumor progression and the development of tumor heterogeneity [11]. The heterogeneity of BTCs manifests at multiple levels, including intertumoral diversity (e.g., distinct molecular subtypes such as IDH1-mutant iCCA or TP53-mutant GBC) and intratumoral heterogeneity driven by clonal evolution and microenvironmental interactions [12, 13]. Interactions between tumor cells and the TME further contribute to heterogeneity, facilitating tumor progression [14]. For example, BTCs can convert nearby fibroblasts into cancer-associated fibroblasts (CAFs), which release cytokines and growth factors that drive tumor growth and metastasis [15]. At the same time, the intricate cellular networks within the TME, including immunosuppressive macrophages and CAFs collaboratively establish an immune-evasion niche via cytokine secretion and extracellular matrix remodeling [16, 17]. These cells achieve this by secreting cytokines, remodeling the extracellular matrix, and engaging in metabolic competition, which contribute to the progression and worsening of the disease [18]. Moreover, tumor cells can adjust their metabolic pathways and invasive capabilities by manipulating the availability of oxygen and nutrients within the TME [19, 20]. The dynamic remodeling of tumor heterogeneity and TME plays a crucial role in determining the therapeutic responsiveness of BTCs, often contributing to drug resistance and treatment failure [21–23].

**Fig. 1:**
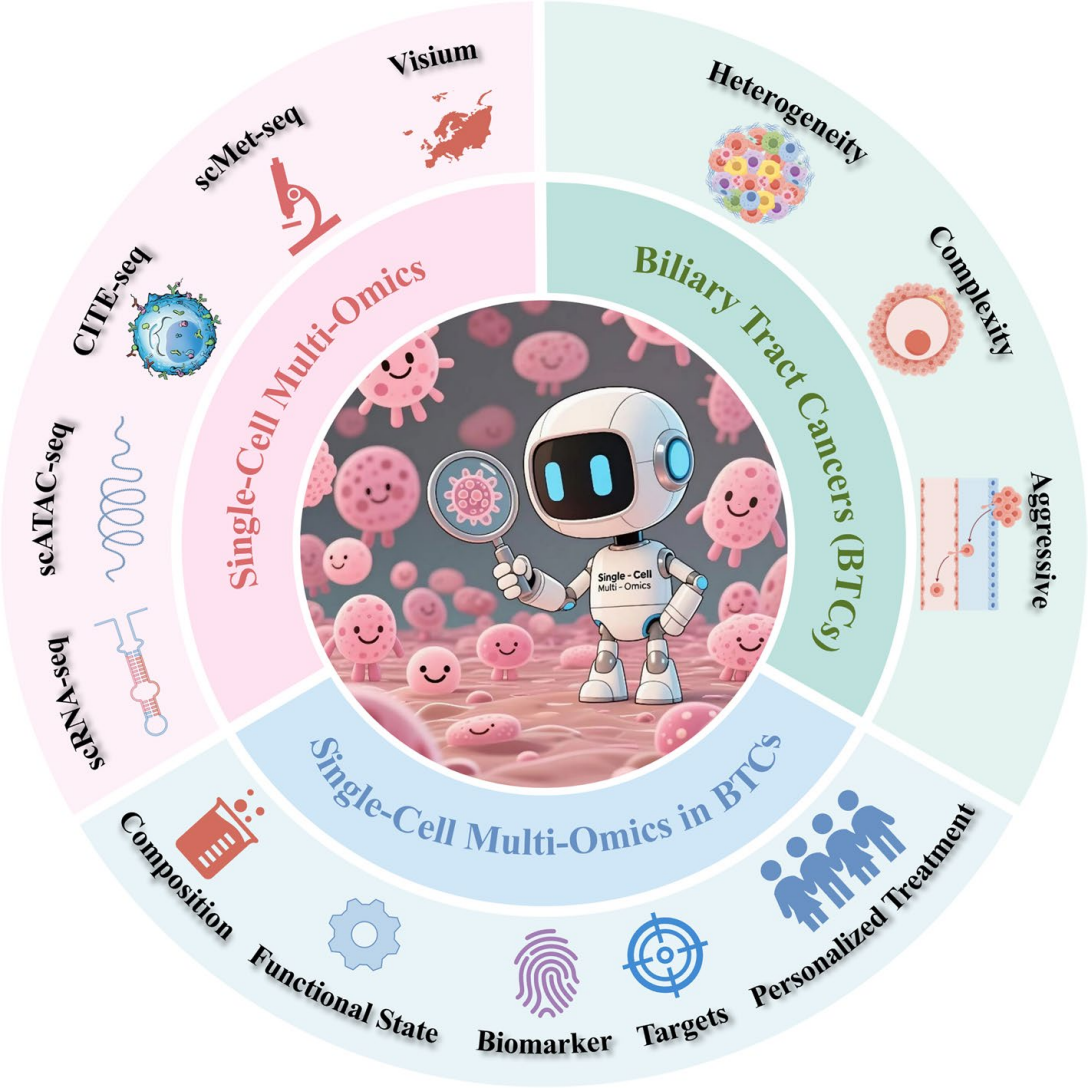
Single-cell multi-omics in BTCs

Recent advancements in integrated single-cell multi-omics technologies such as single-cell transcriptomics, epigenomics, and spatial transcriptomics have opened new avenues for comprehensively understanding BTC heterogeneity, TME remodeling, and their intricate interactions at single-cell resolution [24, 25]. In a proteogenomic analysis of 262 patients with intrahepatic cholangiocarcinoma (iCCA), Fan et al. integrated genomic, transcriptomic, proteomic, and phosphoproteomic data. This in-depth multi-omics approach not only revealed aflatoxin-associated mutational signatures specific to the Chinese population but also classified iCCA into four distinct proteomic subtypes: inflammatory, stromal, metabolic, and differentiated. These subtypes exhibited significant differences in prognosis, mutational landscape, pathway enrichment, and immune microenvironment features. Furthermore, the study identified prognostic biomarkers such as HKDC1 and SLC16A3 [26]. At the same time, spatial transcriptomics has highlighted the localized activation of critical signaling pathways at the tumor-stroma interface [27]. By integrating the analysis of epigenetic and transcriptional regulatory networks within tumor cells and immune matrices, researchers have uncovered the link between APOBEC mutation patterns and immune evasion, as well as the regulatory role of CAF subtypes (such as pro-fibrotic myCAF) in mediating resistance to immunotherapy [28, 29]. Thus, a thorough exploration of the advancements in single-cell multi-omics research for BTC is essential for gaining a deeper understanding of the biological complexity of tumors and as a critical pathway to overcoming clinical treatment barriers and advancing the field of precision medicine (Fig. 1).

While single-cell multi-omics has revolutionized the characterization of breast and lung cancers, its application in BTCs remains in its infancy, with a notable lack of comprehensive reviews to guide future research [30]. This reviewfocus herein on nascent but compelling evidence that motivates the integration ofmultiple strata of information in single BTCs cells todecipher tumour heterogeneity, microenvironment remodeling and evolution to integrate single-cell genomic, transcriptomic, and epigenomic data. Importantly, it also explores personalized treatment strategies informed by single-cell technologies. These findings underscore the importance of single-cell technologies as vital tools for unraveling the complexity of BTCs and driving mechanism-based therapeutic innovations. However, it should be noted that research on AVC is still limited and is not covered in this review.

## The foundational overview of single-cell multi-omics technologies

**Fig. 2:**
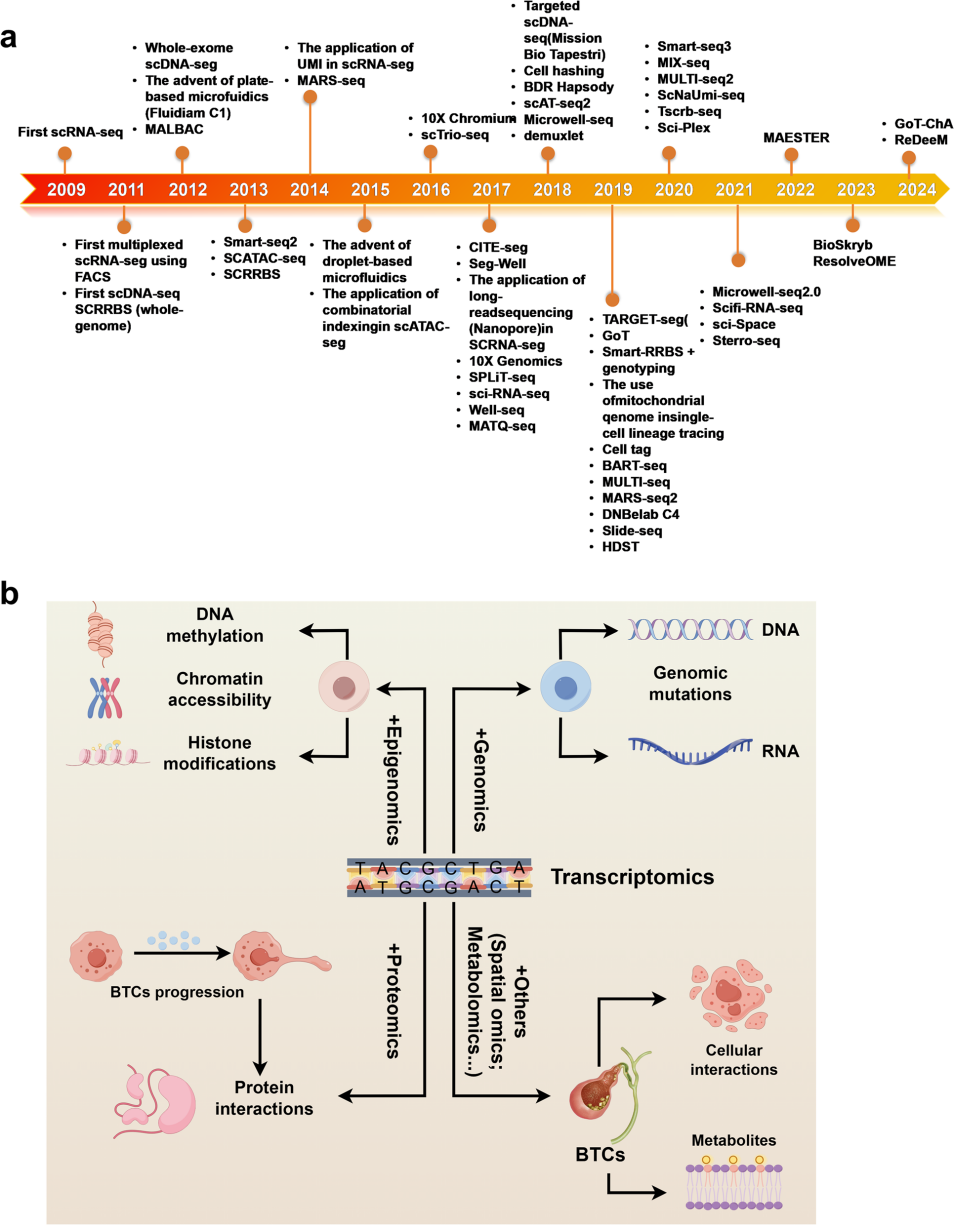
Timeline of single-cell multi-omics technology development and its application in BTCs research. (**a**) Timeline of key single-cell multi-omics technologies from 2009 to 2024, including: transcriptomics, genomics, epigenomics, multimodal assays, spatial transcriptomics/genomics. (**b**) Schematic representation of how multiple molecular layers are integrated inBTCs studies

The advent of single-cell multi-omics technology stemmed from the realization that traditional "bulk" analysis could not capture cellular heterogeneity. Early single-cell studies were constrained by technical limitations, only allowing for observing a few marker proteins using microscopy or flow cytometry [31]. The groundbreaking achievement of scRNA-seq by Tang et al. in 2009 marked the beginning of the single-cell resolution omics era [32]. With technological progress, single-cell technologies have evolved from the use of microfluidic systems and molecular tags (such as UMIs) between 2011 and 2015 (e.g., Fluidigm C1, Smart-seq2) to the current high-throughput commercial platforms, such as 10 × Genomics and BD Rhapsody [33–35]. The significant reduction in the cost of single-cell sequencing has facilitated its transition from basic research to clinical applications. Traditional bulk omics approaches (e.g., bulk RNA-seq, whole-exome sequencing) have provided foundational insights into BTCs biology but are fundamentally constrained by their inability to resolve cellular heterogeneity [36]. These methods average signals across diverse cell populations, masking rare but critical subsets such as tumor-initiating cells, immune evasion niches, or spatially restricted stromal interactions [25, 37]. As a result, critical driving mechanisms and potential therapeutic targets have remained elusive for an extended period. Recently, single-cell technologies have broadened beyond the transcriptome to include the epigenome (scATAC-seq), proteome (CITE-seq), metabolome (scMet-seq), and spatial omics (Visium, MERFISH), enabling multi-dimensional analysis of cellular states [38]. This significant advancement has greatly improved the ability to study individual cells at the transcriptional level, offering new insights into the fundamental processes driving cancer development previously obscured in bulk samples (Fig. 2a).

Single-cell multi-omics integrates multiple modalities to characterize cell states, activities, and heterogeneity, capturing both individual cells and their surrounding microenvironments [39]. This comprehensive profiling is crucial for understanding tumor growth, progression, and responses to therapy [40, 41]. For example, combining single-cell epigenome analysis with transcriptomics allows for directly examining DNA methylation, chromatin accessibility, and histone modifications in conjunction with the corresponding transcriptomic profiles [42–44]. Furthermore, concurrent genome and transcriptome analysis within the same single cell reveals the transcriptional effects of genomic mutations, uncovering novel gene expression patterns linked to diseases [45]. Similarly, integrating the transcriptome and proteome enables the capture of post-translational modifications and protein interactions at the single-cell level, offering a more comprehensive understanding of functional cellular processes [46]. Over 20 distinct single-cell multi-omics technologies have been developed, incorporating a range of integration methodologies. These technologies can generally be divided into two main groups [33, 41, 47]. The first group emphasizes molecular detection, which involves identifying various molecular characteristics of cells in specific states. The second group integrates molecular detection with functional research, allowing for the investigation of changes in gene regulatory networks within cells in response to external perturbations [48]. These approaches improve the thorough and systematic characterization of cellular states and fates and have been extensively applied in diagnosing and treating a range of diseases, including cancer [49, 50]. Moreover, integrating single-cell omics with spatial omics technology preserves the spatial information of cells within tissues, enabling the study of cellular interactions in their native environments [51–53]. Combining data from multiple omics platforms mitigates the limitations of single-omics approaches, allowing for a more comprehensive analysis of gene regulatory networks. Recent research has demonstrated that emerging computational tools, such as Seurat and MOFA^+^, facilitate the integrated analysis of multi-omics data, enabling deeper exploration of their biological significance [54–56] (Fig. 2b).

## Decoding tumor heterogeneity in BTCs through multi-omics

Tumor heterogeneity, a defining characteristic of BTCs, results from molecular and genetic changes throughout tumor evolution, giving rise to variations in growth rates, invasive potential, drug sensitivity, and responses to therapy among different tumor cell populations [57, 58]. This heterogeneity profoundly influences diagnostic accuracy, therapeutic effectiveness, disease monitoring, drug resistance, and overall prognosis [59–61]. Single-cell multi-omics technologies have revolutionized our ability to dissect this complexity, revealing distinct molecular subtypes, clonal evolutionary trajectories, and functional cell states that were previously obscured by bulk sequencing approaches. Below, we present a comprehensive overview of these studies, with selected ones also listed in Table 1.

**Table 1:** Overview of single-cell multi-omics studies in BTCs

Cell Type	Key findings	Functional phenotypes	Potential value	Ref
**Tumor cells**	Intratumoral heterogeneity with distinct clonal evolution patterns	Identify tumor subsets with stem cell characteristics (e.g., high expression of CD44)	Targeting CSC pathways (WNT/Notch inhibitors) to prevent recurrence	[83]
	Active EMT programs	EMT transitional state cells (positive for some EMT markers)	Blocking EMT (e.g., TGF-β inhibitors) to suppress metastasis	[84]
	Metabolic reprogramming (e.g., glycolysis/OXPHOS upregulation)	High-metabolic subclones	LDHA inhibitors to sensitize tumors to chemotherapy	[85]
**Immune cells**	T-cell exhaustion (PD-1, CTLA-4 and LAG3 were highly expressed)	Exhausted CD8^+^ T cells	Combined immune checkpoint inhibitors (e.g., anti-PD-1 anti-CTLA-4) may restore T cell function	[86]
	Enrichment of TAMs	M2-like TAMs (promoting immune evasion)	CSF1R inhibitors or repolarization to M1 phenotype	[87]
	Increased regulatory T cells (Tregs)	Tumor-specific Tregs (high expression of ICOS and CTLA4)	Targeting Tregs may enhance the immune response	[88]
**Stromal cells**	Heterogeneous CAFs	CAF subsets secreting IL-6 and HGF (promoting tumor growth)	Targeting CAF-derived factors (IL-6R or HGF/c-MET inhibitors)	[89]
	Aberrant angiogenesis	Tip cells (migratory endothelial cells)	Anti-angiogenic therapy (VEGFR inhibitors) + immunotherapy	[90]
**Hybrid populations**	Immune-tumor hybrid cells	Double-positive cells (co-expressing epithelial/immune markers)	Bispecific antibodies or CAR-T against hybrid antigens	[91]
	Rare progenitor-like cells	Progenitor-like clusters	Differentiation therapy or early interception	[92]

CSC, Cancer Stem Cell; EMT, Epithelial-Mesenchymal Transition; TGF-β, Transforming Growth Factor-beta; OXPHOS, Oxidative Phosphorylation; LDHA, Lactate Dehydrogenase A; CTLA-4, Cytotoxic T-Lymphocyte-Associated protein 4; LAG3, Lymphocyte-Activation Gene 3; TAMs, immunosuppressive macrophages; CSF1R, Colony-Stimulating Factor 1 Receptor; Tregs, regulatory T cells; CAFs, Cancer-Associated Fibroblasts; CAR-T, Chimeric Antigen Receptor T-cell therapy

### Multi-omics reveal molecular subtypes and genomic alterations

Single-cell multi-omics have systematically revealed the functional status and spatial distribution characteristics across different anatomical subtypes of BTCs (iCCA, eCCA, GBC). Genomic studies have identified key driving events, including IDH1/2 mutations, FGFR2 fusions, and KRAS/BRAF activation. However, these mutations show significant distributional differences across anatomical sites (iCCA vs. GBC) and patient subgroups [9, 62–64]. For instance, FGFR2 fusion is more prevalent in iCCA (approximately 15%), whereas TP53 mutations are found in over 50% of GBC cases [65, 66]. Xiang et al. demonstrated that the IDH mutant subgroup status, rather than the mere presence of an IDH mutation, is associated with intertumoral heterogeneity and the TME [67]. Furthermore, Farshidfar et al. identified IDH-mutant CCA as an independent subtype by integrating genomic, transcriptomic, and epigenomic data. This subtype is characterized by high expression of mitochondrial genes (such as those in the ETC complex), low expression of chromatin modification genes (like ARID1A), and DNA hypermethylation. IDH-mutant CCA shares epigenetic features with certain hepatocellular carcinomas (HCC), indicating potential molecular similarities across different cancer types [68]. Although genomic studies have identified driver mutations such as IDH1, FGFR2, and KRAS, their clinical benefits remain limited. Song et al. further classified iCCA into two subtypes, S100P^+^SPP1^−^ (iCCAPhl) and S100P^−^SPP1^+^ (iCCAPps). The iCCAPhl subtype originates from large bile ducts and is highly invasive and associated with a poor prognosis. In comparison, the iCCAPps subtype, originating from small bile ducts, demonstrates a higher degree of differentiation and a better prognosis. This marker-based subtype classification (S100P/SPP1) aligns with histological classification, and single-cell data have revealed the regulatory role of the CREB3L1 transcription factor in S100P expression [69]. In a pioneering effort, Vindhya Vijay et al. constructed a multi-omics holographic landscape encompassing 63 BTCs cell lines. By integrating whole-exome sequencing, transcriptomic, and proteomic analyses, they not only validated the molecular similarity between cell lines and primary tumors but also systematically mapped genetic dependencies in BTCs, pinpointing critical pathways such as EGFR and KRAS [70]. These highlights novel therapeutic targets that cannot be revealed through genomics alone.

### Multi-omics reveal tumor evolution and clonal dynamics

Through multi-omics approaches, research into tumor evolution and clonal dynamics has uncovered the intricate mechanisms underlying intratumoral heterogeneity and clonal development. Research has shown that early clonal events in iCCA, including FGFR2 fusions, TP53 mutations, and chromosome 6q deletions, frequently accompany gene loss in antigen presentation, such as HLA class I molecules, facilitating immune evasion. Moreover, the extent of immune cell infiltration critically influences clonal evolution patterns: tumors with high immune infiltration tend to show lower clonal heterogeneity due to T cell exhaustion and impaired antigen presentation, whereas those with low infiltration escape immune surveillance by losing subclonal antigens, often through copy number loss of clonal neoantigens. Tumors harboring FGFR2 mutations or fusions are predisposed to an 'immune-cold' phenotype, likely driven by early clonal dominance, low mutational burden, and reduced immune engagement [71].

Furthermore, using multi-region sequencing, Lin et al. showed that intratumoral heterogeneity in iCCA is shaped by parallel evolution and chromosomal instability (CIN). While trunk mutations, such as those in IDH1 and KRAS, were consistently present across tumor regions, branch mutations like TP53 and SMARCB1 were linked to subclonal adaptation, underscoring the therapeutic significance of targeting early, trunk-level alterations [72]. Similarly, Dong et al., demonstrated that clonal evolution in iCCA is driven by CIN and parallel evolutionary processes. Foundational trunk mutations such as those in IDH1 and KRAS were consistently detected across all subclones. In comparison, branch mutations, including TP53 and SMARCB1, were linked to adaptive heterogeneity in specific tumor regions, particularly in cases from Yakron [73]. Meanwhile, spatial transcriptomics and multi-region sequencing have demonstrated substantial inter- and intra-patient variability. For example, Pirenne et al. applied spatial transcriptomics to analyze distinct lesion areas ranging from normal epithelium to low- and high-grade BilIN and adenocarcinoma within the same gallbladder adenocarcinoma tissue. While transcriptomic variation among lesions within individual patients was relatively modest, the pathways of tumor development differed markedly between patients. In one case, adenocarcinoma arose from high-grade BilIN, whereas in another, it progressed directly from low-grade BilIN, highlighting patient-specific trajectories of tumor evolution [74]. A genomic study by Lin et al. further substantiated these findings by identifying two distinct evolutionary trajectories in GBC: the conventional 'adenoma-carcinoma' sequence and an alternative BilIN-independent early branching pathway. CTNNB1 mutations were found to play a pivotal role in both routes. These studies indicate that the heterogeneity of biliary tract tumors arises from spatial distribution differences and dynamic evolutionary processes driven by clonal selection pressures, underscoring the need for therapeutic strategies targeting trunk mutations to achieve broader coverage across tumor subclones. Another study on GBC revealed that the enrichment of exhausted CD8^+^ T cells and Tregs is associated with immune evasion. Based on T cell characteristics, GBC was classified into three types: Type 1, primarily characterized by M2 macrophage infiltration; Type 2, defined by an immunosuppressive microenvironment with exhausted CD8^+^ T cells, B cells, and Tregs; and Type 3, characterized by reduced T cell infiltration and increased immune escape. These findings highlight potential immunotherapy targets for GBC, such as M2 macrophages in Type 1 or exhausted T cells and Tregs in Type 2 [75].

### Multi-omics reveal epigenetic and metabolic heterogeneity

Single-cell multi-omics has been instrumental in deciphering the complex interplay between epigenetic regulation, metabolic reprogramming, and immune evasion that underpins BTC heterogeneity. Qiu et al. discovered that the m6A demethylase ALKBH5 promotes PD-L1 mRNA expression by increasing its stability and suppressing T-cell activity [76]. Similarly, single-cell mass spectrometry analysis revealed that iCCA patients with elevated ALKBH5 expression showed increased sensitivity to anti-PD-1 therapy, highlighting a direct link between epigenetic regulation and immunotherapy responsiveness [77]. Metabolically, specific glycolytic-related cellular subsets (e.g., Epi_SLC2A1, CAF_VEGFA, Mph_SPP1) in CA19-9 + iCCA patients form a synergistic network promoting EMT and angiogenesis [78]. Furthermore, integrated analysis has revealed compensatory mechanisms within the TME; upon targeting tumor-associated macrophages (TAMs), granulocytic myeloid-derived suppressor cells (G-MDSCs) expand via CXCl2-mediated recruitment. G-MDSCs exhibit a STAT1/NF-κB-driven immunosuppressive phenotype, characterized by the downregulation of pro-apoptotic genes like ApoE [79]. Consequently, single-cell multi-omics reveals that within a single tumor, coexisting clones can exhibit distinct characteristics—metabolic reprogramming (IDH1-mutant), inflammation (NF-κB-high), and immune escape (PD-L1^+^)—which collectively contribute to therapeutic resistance and recurrence risk [77, 80–82].

## Remodeling of the tumor microenvironment: multi-omics perspectives

In cancer research, single-cell multi-omics enables the dissection of TME complexity, identifying crucial cell subpopulations such as tumor stem cells and immune cells and revealing gene expression profiles at single-cell resolution, offering valuable insights into potential pathways for precision cancer therapy [93–95]. BTCs demonstrate substantial diversity in their TME profiles, marked by differences in genomic and epigenomic landscapes and other intrinsic characteristics of cancer cells [96]. Moreover, metabolites, cytokines, and growth factors in the BTC microenvironment actively influence tumor cell behavior, contributing to the development of drug resistance [97–99]. Thus, single-cell multi-omics enables monitoring dynamic TME changes during treatment, providing real-time insights into therapeutic effectiveness and facilitating the prediction of drug resistance within the tumor [100, 101]. It is crucial to describe the characteristics of TME.

### Multi-omics reveal immune landscape and evasion mechanisms

Single-cell multi-omics approaches have substantially advanced our understanding of the immunosuppressive landscape in CCA. Single-cell sequencing combined with CellPhoneDB analysis revealed the formation of an immunosuppressive network between Tregs, CD8^+^ Tex, and M2 macrophages through ligand-receptor interactions such as CCL4-CCR8 and HLA-E-KLRK1, suggesting that these interactions may contribute to tumor immune escape [88]. Furthermore, eCCA is characterized by an abundance of exhausted T cells and mature tertiary lymphoid structures (TLS), suggesting a higher sensitivity to immune checkpoint inhibitors. In comparison, iCCA shows a significant expansion of Treg cells and high expression of the immunosuppressive molecule LGALS1. Moreover, when combined with spatial transcriptomic analysis, the study further validated the differences in CD8^+^ T cell density and immunophenotype among subtypes, revealing the subtype-specific immune microenvironment architecture [102]. On the other hand, Bao et al. employed proteomic and single-cell sequencing to classify iCCA into three subtypes: chronic inflammation, metabolic, and chromatin remodeling. Among these, the chronic inflammation subtype—associated with the poorest prognosis—was marked by enrichment of APOE⁺C1QB⁺ macrophages, underscoring the pivotal role of an immunosuppressive TME [103]. Macrophage polarization (M1/M2) and PD-L1 expression further fine-tune immunosuppression: PD-L1⁺ M2 macrophages drive T cell exhaustion, whereas a PD-L1⁻ M2 subset may retain anti-tumor potential [104]. Similarly, the PD-L1-M2 subset potentially showed anti-tumor activity [105]. This finding complements the study by He et al. in GBC [106]. In comparison, GBC induces an aggressive, immunosuppressive environment characterized by TAMs, Tregs, CD8^+^ Tex, and STMN1^+^ CAFs. A subset of senescence-like fibroblasts, enriched in metastatic lesions, promotes GBC migration and invasion through their secretory phenotype [107]. Similarly, Lu et al. showed that neutrophils within the liver metastasis microenvironment of GBC significantly contribute to the increased proliferation, migration, and invasion of GBC cells [108]. Wang et al. emphasized several important discoveries: adenocarcinoma cells could transdifferentiate into squamous tumor cells, macrophages in GBC tissue transitioned from pro-inflammatory to anti-inflammatory states, and mesenchymal and endothelial cells played an active role in promoting angiogenesis and lymphangiogenesis, both crucial for sustaining tumor growth and metastasis [106, 109]. Mechanistically, Liang et al. revealed that HIF1A activation in iCCA promotes TAM polarization toward M2-like phenotypes via the PPARG–CD36 axis and recruits Tregs through CCL3–CCR5 signaling, fostering an immunosuppressive niche [110]. Furthermore, Ruan et al. elucidated a vicious cycle in iCCA wherein YAP-driven tumor cell dedifferentiation is modulated by TAMs, which in turn are polarized toward M2 states via tumor-derived IL-6 and TGF-β. Disrupting TAMs interrupted this circuit and enhanced T cell responses, suggesting a promising combinatory therapeutic strategy [89].

### Multi-omics reveal intercellular interactions in the TME

The interstitium of the immune microenvironment (IME) in patients with BTCs presents a dense fibrotic feature, with a large amount of CAFs and constituting the main cell population of the interstitium [111]. At present, the understanding of its anti-tumor and anti-tumor immune effects in research is still limited. The CAFs in the IME can directly interact with biliary tract tumor cells or indirectly recruit immunosuppressive cells by generating multiple factors, and plays a key role in the occurrence and progression of BTCs.

As the most prevalent mesenchymal cells in the TME, the heterogeneity and functional plasticity of CAFs have been highlighted in numerous studies. Affo et al. noted that in iCCA, CAFs exist in both tumor-promoting and tumor-suppressing subgroups, with their phenotypes undergoing dynamic changes throughout tumor progression. scRNA-seq analysis shows that CAFs establish a complex interaction network with tumor cells, endothelial cells, and immune cells (such as TAMs and Tregs) via signaling pathways like TGF-β and PDGF-D, thus contributing to fibrosis and immunosuppression [112]. This finding aligns with identifying the interstitial ecological subtype SC1 (Fibro-iCAF and Endo-Tip interaction) and the dynamic polarization trajectory of immunosuppressive APOE^+^ macrophages in GBC. Specifically, the spatial transcriptomics study by Li et al. further revealed the unique microstructure of the leading-edge region of the tumor: highly proliferative tumor cells form a "trisomy structure" with POSTN^+^ FAP^+^ CAFs, SPP1^+^ macrophages, and endothelial cells, which together promote tumor invasion through mechanical signals and paracrine signals. In this region, CD8^+^ T cells demonstrate an exhausted phenotype (high PD-1, low GZMB), while MAIT cells enhance immunosuppression by recruiting SPP1^+^ macrophages. This suggests that the interaction between CAFs and immune cells facilitates immune escape [113]. Furthermore, CAF participates in the invasion of CCA cells by secreting various matrix metalloproteinases [114]. In the mouse model with CAF gene deletion, the growth of CCA cells was also inhibited [115]. In addition to directly influencing tumor cells by regulating their proliferation, survival and migration, CAFs regulate angiogenesis and immune growth factors by secreting a large amount of soluble chemokines. Chemokines such as C-X-C motif ligand 14 (CXCL14), interleukin-8 (IL-8), IL-13, vascular endothelial growth factor (VEGF), and fibroblast growth factor (FGF) stimulate stromal cells and recruit inflammatory immune cells in the tumor bed [116]. Sun et al. found that the cellular communication between malignant cells and CAFs was significantly enhanced, especially promoting angiogenesis through the VEGF signaling pathway mediated by FGF-VEGFR1, suggesting the possible value of targeted CAFs combined with anti-angiogenic therapy [117]. Shi et al. discovered that histamine promotes tumor proliferation and angiogenesis by differentially activating CCA cells (via HRH1-Gαq signaling) and CAFs (via HRH2-Gαs signaling).

**Fig. 3:**
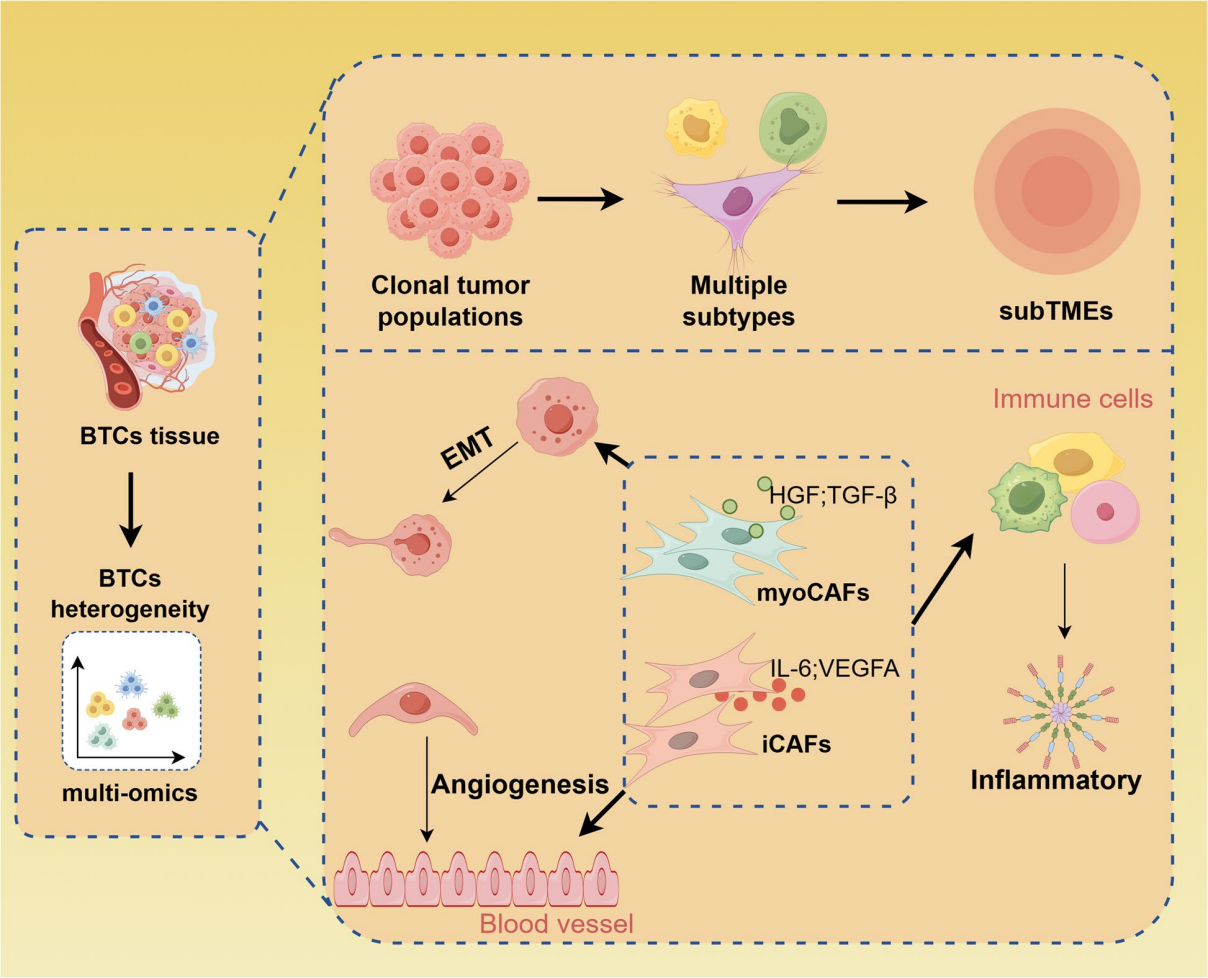
Single-cell multi-omics reveals tumor heterogeneity and microenvironment interactions in BTCs. The diagram highlights: clonal tumor populations comprising multiple molecular subtypes, contributing to BTCs heterogeneity. Distinct TME components, including: cancer-associated fibroblasts (CAFs) differentiated into: myoCAFs (promoting angiogenesis via HGF and TGF-β signaling); iCAFs (secreting inflammatory cytokines such as IL-6 and VEGFA); Immune cells involved in inflammatory responses

Moreover, Ravichandra et al. examined CAF heterogeneity in iCCA using single-cell sequencing. They identified multiple functional subgroups, including myCAF (high expression of α-SMA and COL1A1), iCAF (high expression of IL-6 and CXCL12), and apCAF (expression of HLA-DR antigen presentation molecules). These subgroups form a dynamic interaction network with tumor cells, endothelial cells, and macrophages by secreting ECM components (such as collagen and POSTN) and growth factors (like HGF and CXCL12). Among these, myCAF increases chemotherapy resistance by promoting ECM hardening, while apCAF may modulate T cell function through antigen presentation [118]. These findings underscore the dual role of CAFs in the TME. Hong et al. classified CCA into three subgroups: estrogen signaling activation (ESTRO), oxidative phosphorylation activation (OXPHO), and immune pathway activation (IMMUN) based on an integrated multi-omics analysis of enhancer activity [119]. The IMMUN subtype is marked by a high tumor mutational burden (TMB) and significant immune cell infiltration; however, the response to immune checkpoint inhibitors varies widely, partially due to the spatial heterogeneity of CAFs. Spatial transcriptomics revealed that regions with a high aristolic acid (AA) mutant burden were co-localized with CD8^+^ T cell exhaustion and elevated POSTN expression in CAFs, suggesting that the mutant background influences the interaction between CAFs and immune cells via epigenetic regulation. This study offers a theoretical foundation for therapeutic strategies targeting the CAF-immune axis, such as combining anti-POSTN and PD-1 inhibitors [81]. These findings highlight the "double-edged sword" role of CAFs in the TME, where they facilitate tumor invasion and hinder therapeutic efficacy by promoting immunosuppression. As central regulators of the TME, the functional diversity of CAFs, ranging from tumor-promoting to tumor-suppressing roles, is closely linked to their metabolic status and epigenetic background. Targeting specific CAF subgroups or blocking key interaction pathways could offer novel strategies for overcoming immunosuppression and therapeutic resistance (Fig. 3).

### Multi-omics reveal immune regulation of metabolic reprogramming

Single-cell multi-omics technology has also facilitated the analysis of the metabolic interaction network between tumor cells and immune cells, offering a molecular foundation for immunotherapy strategies that target metabolism [120]. Deng et al. addressed this issue from a metabolic standpoint and categorized CCA into "active" and "inactive" subtypes based on bile acid metabolism. The "active" subtype is characterized by the upregulation of bile acid metabolism genes (such as CYP7A1 and SLC10A7) and an immunosuppressive microenvironment, including an enrichment of Tregs and M2-type macrophages. The "inactive" subtype shows increased infiltration of CD8^+^ T cells and low expression of immune checkpoint molecules. Single-cell analysis revealed that bile acids regulate the activation of CAFs through FXR receptors, influencing T-cell function [121]. This discovery aligns with the previously discussed CAF-mediated immunosuppressive mechanisms and further highlights the direct link between metabolic reprogramming and the immune status within the TME. Moreover, Lewinska et al. found that lysyl oxidase (LOX) secreted by CAFs drives the metabolic adaptability and stem cell properties of CCA cells by promoting OXPHOS and enhancing mitochondrial function. High LOX expression correlates significantly with KRAS mutations, metastasis, and poorer patient survival. Inhibition of LOX can reduce tumor burden and improve liver function [85].

Sang et al. investigated the role of lactic acid metabolism in CCA and developed a prognostic model based on lactic acid metabolism-related genes (LMRGs). They found that patients with a high LMRG score had poorer prognoses associated with TP53/KRAS mutations, impaired NK cell function, and increased neutrophil infiltration. Lactate dehydrogenase A (LDHA) was identified as a critical molecule. Its elevated expression promotes tumor proliferation via activation of the MAPK/ERK pathway, inhibits NK cell activity, and contributes to an immune escape phenotype. Knockdown of LDHA significantly inhibited tumor growth and reversed the immunosuppressive microenvironment [122]. Chai et al. were the first to analyze the characteristics of the intracellular microbiome in iCCA using single-cell and spatial omics. They discovered that *Paraburkholderia fungorum* was enriched in adjacent tissues and inhibited tumor growth by regulating the metabolism of alanine, aspartic acid, and glutamic acid. The abundance of this bacterium was negatively correlated with CA199 levels, suggesting its potential as a prognostic marker [123]. Zhang et al., through integrated metabolomics and CRISPR/Cas9 screening, identified the central role of the PPARγ-FABP4 axis in lymph node metastasis in CCA. Oleic acid promotes FABP4 expression by activating PPARγ, creating a positive feedback loop that increases the metabolic dependence of tumor cells on fatty acids enriched in lymph nodes, supporting their colonization and proliferation. This finding illuminates the metabolic adaptation mechanisms under spatial heterogeneity and provides a foundation for targeted therapeutic strategies that address the intersection of metabolism and immunity [124]. Furthermore, intratumoral microorganisms influence the immune response by regulating the functions of TAMs and T cells, offering a novel direction for combined therapies targeting microbiota-immune interactions [125].

The studies above highlight the pivotal role of single-cell multi-omics in uncovering the heterogeneity of BTCs and the regulatory mechanisms of immune metabolism, underscoring its potential in precision medicine research. In summary, these studies provide a comprehensive analysis of the dynamic network of the TME, exploring its heterogeneity, spatial interactions, metabolic-immune cross-regulation, and molecular subtyping. And the development of an immune cell atlas not only offers biomarkers for prognostic stratification but also suggests combination treatment strategies targeting specific cell subsets or signaling pathways (e.g., anti-PD-L1 combined with TAM inhibitors) through the analysis of immunosuppressive mechanisms like TAM-M2 polarization and CAFs heterogeneity [126–128]. These studies have outlined the immune profile of BTCs using single-cell multi-omics approach, emphasizing the crucial role of single-cell technology in analyzing tumor heterogeneity, uncovering low-abundance functional subsets, and understanding intercellular communication, thus providing a foundation for targeted therapy in BTCs.

## Translating multi-omics insights into treatment strategies in BTCs

### Multi-omics in targeted treatment

Currently, the use of targeted therapies for BTC is still under exploration, although some targeted drugs have demonstrated promising therapeutic effects in BTCs with specific molecular characteristics. The targeted therapies for BTC approved by the US Food and Drug Administration (FDA) can primarily be classified as IDH1 inhibitors, FGFR-2 inhibitors, and NTRK inhibitors [129]. In addition to these, drugs targeting BRAFV600E mutations, HER2, VEGF/VEGFR, PTEN, the Notch pathway, PARP inhibitors, and sphingosine kinase 2 inhibitors are also in clinical trial stages for BTC [57, 90, 130, 131]. Recent research indicates that HER2 amplification occurs in 10% of patients with advanced BTC. For patients receiving HER2-targeted therapies, such as trastuzumab deruxtecan, the median OS was significantly extended to 24.3 months, compared to 12.1 months for those not receiving the treatment. Although HER2 amplification is not an independent prognostic factor, targeted therapy has significantly improved survival outcomes [154]. A genomic mutation analysis of BTCs in a cohort of 803 Chinese patients revealed multiple actionable targets, including FGFR, IDH, NTRK, HER2, KRAS (Kirsten rat sarcoma viral oncogene homolog), and cyclin E1, offering promising avenues for targeted therapy development [132]. Identifying these targets has expedited the development of clinical trials for targeted therapies. Recently approved FGFR and IDH inhibitors highlight significant progress in treating BTCs [133, 134]. Farshidfar et al. highlighted that IDH mutations lead to the accumulation of 2-hydroxyglutaric acid (2-HG), which inhibits histone demethylases, driving epigenetic dysregulation. This provides a theoretical foundation for the targeted use of IDH inhibitors, such as ivosidenib [68].

Single-cell multi-omics facilitates the discovery of novel therapeutic targets for BTCs. Diagnostic markers in serum extracellular vesicles (EVs)—such as CRP, PIGR, and FIBG—were found to be highly enriched in malignant bile duct cells but scarcely detectable in normal tissues or non-tumor cells [135]. Additionally, LAIR2 and PNOC were identified as markers of T cell exhaustion and B cell anti-tumor activity, respectively, suggesting potential combination immunotherapy strategies, such as anti-PD-1 therapy coupled with LAIR2 blockade [136]. Ni et al. conducted a multi-omics analysis of BTC patients and revealed that mutations in TP53, BRCA2, and cytokine genes, as well as high tumor mutational burden, were significantly associated with treatment response. In contrast, KRAS G12D and ARID1A mutations correlated with poorer survival outcomes. The study also found that high expression of CXCL9 and CTLA4 was linked to improved treatment response, longer progression-free survival, and overall survival. Furthermore, the critical role of CXCL9 in enhancing T cell infiltration and activation was validated through in vivo and in vitro experiments [137]. Furthermore, Da et al. identified CASK as an independent prognostic marker through proteomics. Low expression of CASK is linked to ECM remodeling and immunosuppression, and it may influence the adhesion signaling between CAFs and tumor cells by regulating the integrin pathway [138]. The elevated nuclear expression of ALKBH5 can serve as a predictive marker for the effectiveness of PD-1 inhibitors [77], while patients with low DPT expression may benefit from CCL19 agonists to increase immune infiltration [139]. Morever, Studies has shown that HERVH [140], YKL-40 and GDF15 as promising diagnostic biomarkers and potential targets for immunotherapy in GBC [141]. In conjunction with the transcription factor regulatory network, single-cell multi-omics revealed that USF2 directly binds to the PPP1R1B promoter, driving its expression and activating pro-cancer pathways such as PI3K/AKT. This suggests that PPP1R1B could be a potential therapeutic target for LD-type iCCA [142]. Furthermore, the study emphasized the pivotal role of silencing the solute carrier family 39 members 4 genes (SLC39A4) in iCCA progression, which resulted in increased cell death and the upregulation of key cuproptosis-related genes, such as dihydrolipoyl transacetylase (DLAT) and ferredoxin 1 (FDX1) [143]. Considering the altered copper metabolism and elevated copper requirements in cancer cells, copper-induced apoptosis may contribute to the pathogenesis of iCCA, providing additional opportunities for identifying novel therapeutic targets [144] (Table 2).

**Table 2:** Overview of biomarkers of single-cell multi-omics analysis of BTCs

Samples	Biomarkers/Targets	Ref
4 GBC patients	HERVH	[140]
9 GBC patients	YKL-40 and GDF15	[141]
15 GBCs, 4 cholecystitis samples, 3 gallbladder polyps, 5 gallbladder adenomas and 16 adjacent normal tissues	OLFM4	[145]
2 GBC patients Number of ROIs subjected to spatial transcriptomic analysis	SEMA4A	[74]
10 ICC patients from GSE125449 and 255 ICC patients	SPP1	[9, 146, 147]
8 ICC patients from the GEO database	SLC16A3, TUBA1B, A2M, CEBPB, PMAIP1, CLEC11A, TPM2, BNIP3L, and EREG	[148]
EVs from patients with isolated PSC (n = 45), concomitant PSC-CCA (n = 44), PSC who developed CCA during follow-up (PSC to CCA; n = 25), CCAs from non-PSC aetiology (n = 56), and hepatocellular carcinoma (n = 34) and healthy individuals (n = 56)	CA19-9	[135]
8 ICC patients	EZH2	[149]
3 ICC patients tumor tissue and blood	APOE^+^C1QB^+^	[103]
TCGA CHOL, GSE32225, and GSE26566	LAIR2 and PNOC	[136]
32 ICC patientss from the TCGA database	SLC39A4	[143]
132 ICC patients	PPP1R1B	[142]
mouse ICC tissues	HES1, CFL1, and ID1	[150]

ROIs, Regions Of Interest; PSC, Primary Sclerosing Cholangitis; GEO, Gene Expression Omnibus; EVs, Extracellular Vesicles; CHOL, Cholangiocarcinoma

### CRISPR-integrated multi-omics predict therapeutic targets

In recent years, CRISPR screening combined with single-cell multi-omics has enabled the identification of key genes (such as immune checkpoints or cytokine-related factors) that regulate the immunosuppressive TME. Single-cell omics can further clarify the role of these target genes in specific cell subpopulations. For example, Xu et al. used genome-wide CRISPR screening to uncover that ELP5 influences the sensitivity of GBC to gemcitabine by regulating the Elongator complex and the hnRNPQ/P53 axis [151]. Younger et al. further integrated CRISPR screening with computational analysis to explore the relationship between genetic heterogeneity and core signaling pathways in cholangiocarcinoma. Their study found that, despite significant variation in mutation profiles among patients, the co-activation of the Wnt and PI3K pathways is a shared dependency for tumor growth. This research suggests a cross-patient therapeutic strategy focused on the combined inhibition of Wnt/PI3K [152]. Similarly, Liu et al. employed CRISPR to knock out RBM39, coupled with RNA-seq and drug inhibitors. They uncovered that RBM39 activated the Wnt7B/β-catenin pathway by regulating the splicing activity of EZH2, promoting the progression of CCA. The study further demonstrated the synergistic anti-tumor effects of the RBM39 inhibitor Indisulam and the EZH2 degrader MS177, underscoring the importance of CRISPR screening in target validation and combination therapy development [149]. Xu et al. created the first spontaneous cHCC-ICC mouse model and, through transcriptome analysis, identified LAMB1 as a potential therapeutic target [153]. This model offers a platform for investigating tumor-stroma interactions within the TME, and the integration of CRISPR technology in such complex models aids in simulating patient-specific mutations and assessing their effects on immune infiltration. In conclusion, the combined use of CRISPR screening and multi-omics technologies not only enhances the understanding of biliary tract tumor heterogeneity and the dynamic remodeling of the TME but also paves the way for discovering cross-patient immunotherapy targets and designing combination treatment strategies.

### Multi-omics in mechanisms of immune checkpoint inhibitors

During the progression of BTCs, tumor cells release immunosuppressive factors that establish an immunosuppressive microenvironment, facilitating immune evasion and promoting tumor growth and metastasis. Immunotherapy seeks to modulate the immune system to control and eliminate tumor cells, employing immune checkpoint inhibitors (ICIs), adoptive cell therapy, and tumor vaccines. Zheng et al. demonstrated that the median overall survival (OS) with first-line ICIs combined with chemotherapy was 15.7 months, significantly better than the 9.8 months seen with second-line treatment. However, the effectiveness of different ICIs (e.g., Durvalumab vs. Sintilimab) can vary [154]. The Phase III KEYNOTE-966 trial confirmed that Pembrolizumab combined with chemotherapy helps maintain patients' health-related quality of life (HRQoL), supporting its use as the first-line standard treatment [155]. Furthermore, studies have shown that the abnormal activation of the PD-1/PD-L1 pathway in BTCs can induce immune tolerance by suppressing T cell function [156]. At the same time, the co-expression of other co-inhibitory molecules, such as CTLA-4 and LAG-3, further worsens T-cell exhaustion [146, 157–159]. It is important to note that single-cell sequencing technology uncovers the high heterogeneity of the TME in BTCs, with immunosuppressive subsets such as TAMs, Tregs, and MDSCs undergoing dynamic changes in both spatial distribution and functional status [11, 160]. The cytokines they secrete, such as TGF-β and IL-10, can remodel the extracellular matrix, creating a physical immune barrier [161]. Moreover, the genomic instability of tumor cells may indirectly impact the efficacy of ICIs by modulating the antigen presentation mechanism or interfering with the interferon signaling pathway [162–164]. A comprehensive analysis of the multi-dimensional interaction network between immune checkpoint molecules and TME components in BTCs will provide crucial theoretical insights for optimizing immunotherapy strategies. In addition to PD-1/PD-L1, TIGIT and CD73 have emerged as key immunosuppressive molecules in BTC. TIGIT is highly expressed in the exhausted CD8^+^ T cells of eCCA, with positive cells demonstrating a PD-1^+^TIM-3^+^ phenotype, which is negatively correlated with patient survival rates. Monotherapy targeting TIGIT significantly boosted CD8^+^ T cell activity and inhibited tumor growth in the PDX model [86].

Immunotherapy has emerged as a promising strategy for treating BTCs as our understanding of the immune microenvironment improves. The introduction of ICIs, such as PD-1/PD-L1 inhibitors, has transformed the treatment landscape for various cancers. Research continues to make significant strides in adapting these approaches to address advanced BTCs.

### Multi-omics in personalized treatment

**Fig. 4:**
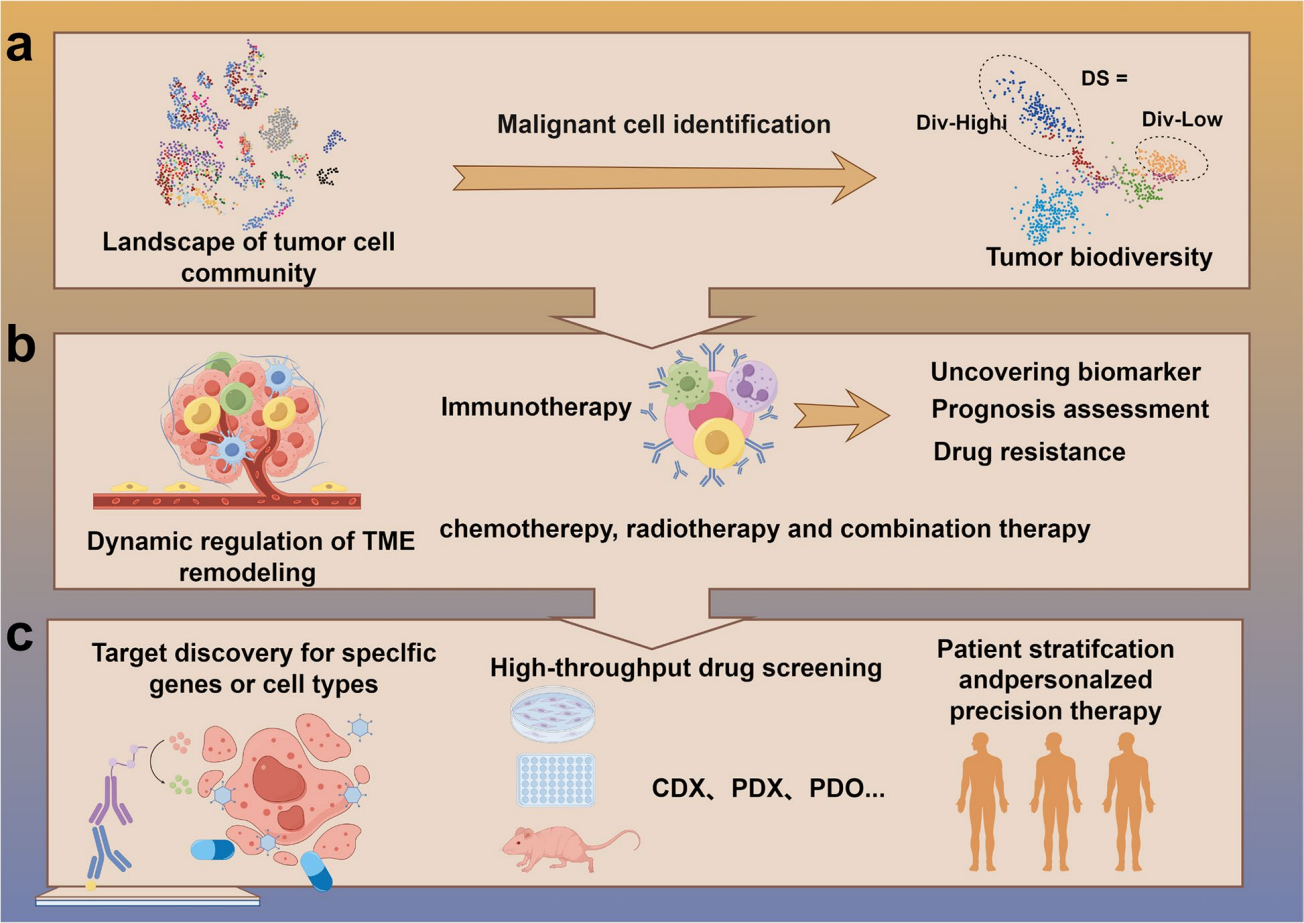
The clinical application of single-cell multi-omics in BTCs. **a** Mapping the tumor cell atlas. **b** Personalized treatment for BTCs. **c** Drug target and sensitivity testing of BTCs

The TOPAZ-1 trial, a global multicenter Phase III study, assessed the efficacy and safety of combining durvalumab with standard chemotherapy versus chemotherapy alone as a first-line treatment for advanced BTCs. The primary endpoint, OS, demonstrated a significant survival advantage with combination therapy compared to chemotherapy alone [165]. Moreover, the KEYNOTE-966 trial showed that adding pembrolizumab to gemcitabine and cisplatin significantly prolonged survival in unresectable or advanced BTC patients without introducing new safety signals [166]. These results have marked a shift in the treatment landscape of BTCs, moving from conventional two-drug chemotherapy toward immune-based combination regimens, signaling the onset of a new era in immunotherapy [167]. However, the substantial heterogeneity of BTCs poses significant challenges, as treatment efficacy and tolerability can differ widely across patient populations and tumor subtypes [168]. A retrospective analysis involving four CCA patients treated with carrilizumab offered further insight into the heterogeneity of GBC and its clinical significance [169]. One patient with GBC demonstrated no response to treatment and experienced disease progression. Transcriptomic analysis of the tumor tissue revealed low expression of key immunotherapy-related genes, including markers of microsatellite instability, tumor mutational burden, DNA mismatch repair, and PD-L1. These findings underscore the profound intertumoral heterogeneity in GBC and emphasize the need for personalized treatment approaches tailored to the molecular characteristics of each patient [170, 171]. He et al. combined whole-genome sequencing (WGS) with single-cell RNA sequencing (scRNA-seq) of clinical samples spanning cholecystitis, polyps, adenomas, and GBCs. Their findings revealed that Olfactomedin 4 (OLFM4), originating from malignant epithelial cells, plays a pivotal role in GBC progression. OLFM4 was linked to T-cell dysfunction and increased tumor-associated macrophage (TAM) infiltration, and its elevated expression was associated with poor patient prognosis [145] (Fig. 4a&b).

### Multi-omics in prognosis assessment and drug resistance

Identifying prognostic biomarkers addresses the limitations of traditional clinical staging systems and enables molecular subtype–based risk stratification. Xie et al. revealed that specific immune cell populations within the TME of BTCs are linked to distinct clinical outcomes. Gene set enrichment analysis (GSEA) was employed to assess pathways associated with the infiltration of specific cell subtypes. Research has shown that iCCA shows a distinct immune ecosystem with various cell subtypes. The newly identified subgroups, SPP1^+^ S100P^+^, and MS4A1^−^SPP1^+^ S100P^+^, play crucial roles in the progression and prognosis of iCCA [172]. Secreted phosphoprotein 1–positive (SPP1⁺) TAMs were associated with poor prognosis, while CXCL13⁺ T cells marked a “hot tumor” phenotype characterized by elevated immune scores and a stimulatory immune milieu. This immune-active subtype correlated with improved survival and heightened sensitivity to immunotherapy, suggesting its potential as a predictive biomarker for treatment response in BTCs [173]. Similarly, Ren et al. developed a prognostic model based on the expression of copper apoptosis-associated genes to categorize patients with iCCA. Their study revealed that high CUPT scores were associated with distinct somatic mutation profiles, immune cell infiltration patterns, and chemotherapy responsiveness, offering valuable insights for personalized treatment strategies in iCCA. On the other hand, Zhong et al. developed a CAF infiltration scoring model using genes such as EVA1A and APBA2, integrating extensive single-cell transcriptomic datasets. Their findings revealed that a high CAF score was significantly correlated with poor patient prognosis [174].

As BTCs advance, genetic mutations within tumor cells can lead to differential therapeutic sensitivities. Furthermore, components of the TME, including vasculature, immune cells, and the extracellular matrix, can influence drug delivery and tumor cell responsiveness. Elevated expression of drug-metabolizing enzymes in cancer cells frequently results in accelerated drug inactivation, thus promoting resistance. Cancer cell subsets with stem-like properties can drive chemoresistance by activating signaling pathways such as Notch and WNT [83, 175]. Moreover, tumor cells may develop stem cell-like traits through EMT, which increases their survival and drug resistance capabilities. To counter this, researchers are increasingly investigating innovative therapeutic approaches, such as molecularly targeted treatments, immunotherapies, and combination drug regimens, to overcome resistance and improve clinical outcomes. Huang et al. found that mutations in TP53 and NRAS were linked to resistance to ICB. At the same time, the interaction between DCs and macrophages, mediated by the CXCL signaling pathway, may facilitate immune evasion [176]. In response, Sun et al. confirmed that SPP1^+^ TAMs are an independent predictor of poor prognosis following ICB treatment, suggesting that targeting SPP1 or combining it with CSF1R inhibitors could potentially reverse drug resistance [87]. Similarly, CD73 is overexpressed in the malignant cells of iCCA, enhancing the infiltration of Treg and M2-type TAMs via adenosine signaling while promoting EMT and stem cell-like characteristics, contributing to resistance to chemotherapy and ICIs [177]. Keenan BP and colleagues identified a distinct tumor-driven immunosuppressive mechanism in which monocytes emerging after anti-PD-1 therapy contribute to immune ICI resistance by inducing T-cell paralysis [87]. Yuan et al. showed that ASPH expression is strongly associated with drug resistance to chemotherapy agents like gemcitabine and paclitaxel. Single-cell omics further revealed that ASPH reduces drug sensitivity by activating the cytochrome P450 metabolic pathway [84]. Lapin et al. monitored the clonal evolution of patients with IDH-mutated cholangiocarcinoma using ctDNA. They discovered that patients with a lower baseline ctDNA variant allele frequency (VAF) experienced longer treatment failure times (3.6 vs. 1.5 months). This suggests a link between tumor burden and treatment response. As treatment progressed, new clonal mutations, such as ARID1A and TP53, were identified in ctDNA, reflecting an adaptive drug resistance mechanism. The study confirmed the consistency of ctDNA with tissue detection (sensitivity 84%) and highlighted its potential for real-time monitoring of clonal dynamics and drug resistance evolution in clinical settings [178].

Uson Junior and Borad highlighted that FGFR inhibitors (e.g., Pemigatinib, Infigratinib) and IDH inhibitors (e.g., Ivosidenib) have significantly improved the prognosis of patients by targeting driven clones, but drug resistance remains a major issue. For instance, the response rate of FGFR2 fusion tumors to these inhibitors can reach 35.5%, yet acquired mutations or bypass activation often result in recurrence. The authors emphasize the importance of ctDNA and spatial multi-omics approaches in analyzing clonal evolution and designing combination therapies, such as dual inhibitors or combined immunotherapy, to address FGFR resistance mechanisms [179]. It offers multi-level targets for precise treatment, from subtype-specific mutations and immune microenvironment regulation to matrix-tumor interactions. In the future, integrating liquid biopsy markers and molecular classification will be essential for early diagnosis while overcoming treatment resistance through targeted combination strategies. This approach will facilitate the shift of BTCs from traditional chemotherapy to personalized treatments focusing on microenvironment remodeling and immune regulation.

## Current challenges and future perspectives of single-cell multi-omics

### Limitations

With the rapid advancement of life sciences, researchers are moving beyond traditional population sample analysis and diving into the complexities of individual cells. Single-cell omics, a cutting-edge technology, provide unprecedented opportunities to uncover the composition and functional state of individual cells directly. These technologies clarify the dynamic changes in cells during various biological processes and deepen our understanding of disease mechanisms. However, the broad application of single-cell multi-omics faces several technical challenges, the most significant being analysis sensitivity, sample processing complexity, and the high difficulty of data analysis [180].

Firstly, sample acquisition is challenging, and the quality is often limited: BTCs, such as iCCA, have limited surgical opportunities, resulting in a scarcity of available samples [181]. Furthermore, multi-omics data integration still depends on the optimization of computational algorithms, particularly in cross-modal data alignment and batch effect correction, which are current bottlenecks [182]. Moreover, tumor tissues are frequently abundant in interstitial components, such as fibrotic or inflammatory cells, leading to a low proportion and reduced activity of tumor cells. As a result, single-cell separation may introduce significant deviations [183]. Secondly, there are substantial technical bottlenecks: insufficient sequencing sensitivity may overlook low-abundance transcripts (such as driver genes IDH1/2 or FGFR2 fusions), amplification biases can distort gene expression quantification, and single-cell technology itself cannot capture the in situ spatial information of cells, making it challenging to analyze the spatial regulatory mechanisms within the microenvironment [67, 81]. Furthermore, the complexity of data analysis is considerable: the vast amount of high-dimensional data places strict demands on algorithms and computational resources, and the absence of a biliary-specific cell marker database complicates the precise distinction between malignant cells and normal cells (such as bile duct epithelial cells) or rare subpopulations [184]. The dynamic analysis of tumor heterogeneity and the interaction network within the microenvironment still necessitates improved models, such as those focused on clonal evolution and cell communication analysis [102]. The biological complexity of tumors further complicates the challenge: The dynamic interactions between immune cells, fibroblasts, and tumor cells are not fully understood, and a single sample is often insufficient to capture the spatiotemporal heterogeneity of tumors. Moreover, there are obstacles in translating these findings into clinical practice: high costs hinder large-scale implementation, functional validation of potential targets, and the development of personalized treatment plans remain areas that require further breakthroughs, and both the experimental process and data analysis lack standardization. Issues related to data sharing and ethics also need to be addressed.

### Integration of preclinical model

#### The PDO model revealed drug sensitivity

Patient-derived organoids (PDOs) are three-dimensional (3D) culture models derived from cancer cells that closely mimic the histopathological and genetic features of primary tumors, including those from the colon, pancreas, and liver [185]. PDOs are valuable tools for personalized chemotherapy drug screening, facilitating the development of gene sets that predict chemotherapy responses based on drug sensitivity. This method helps select the most effective treatments for individual BTC patients [186]. PDO models offer deeper insights into tumor biology and drug responses when integrated with single-cell multi-omics sequencing, such as scRNA-seq and scATAC-seq.

The first organoid model of BTCs was established by Saito, who screened 339 clinical drugs and identified 22 effective anti-tumor drugs for the biliary system [187]. Afterward, Wang et al. successfully established organoids from five GBC cases and performed drug sensitivity tests using commonly used clinical chemotherapy drugs. They observed significant variation in drug sensitivity among GBC samples from different patients, which aligns with the previously mentioned characteristic of high intertumor heterogeneity in GBC [188]. Yuan et al. collected surgically resected tumor tissues from 41 untreated GBC patients and 5 untreated GBA patients, developing a 3D culture protocol for gallbladder tumors. The researchers then performed single-cell sequencing and high-throughput drug screening, revealing heterogeneity among GBC patients. They identified the PI3K/HDAC inhibitor CUDC-907, which significantly inhibited the growth and proliferation of GBC organoids [189]. Similarly, Zhao et al. isolated primary tumor samples from four liver cancer patients, two with iCCA and one with GBC, and cultured tumor organoids in vitro. They identified HCC272, an organoid with an EMT phenotype, which showed broad-spectrum drug resistance. UMAP and pseudotemporal analyses revealed intratumor heterogeneity and distinct evolutionary trajectories within hepatobiliary organoids, with CTNNB1, glyceraldehyde 3-phosphate dehydrogenase (GAPDH), and NEAT1 being prominent expression clusters across samples. Further, the combination of GAPDH and NDRG1 was identified as an independent risk factor and a predictor of patient survival [190].

Akitada Yogo et al. established four PDO models (such as Sph18-08 and Sph18-16) from surgical specimens to investigate the effects of DRD1 inhibition on chemotherapy resistance and transplantation ability. The results demonstrated that inhibiting dopamine receptor D1 signaling promotes human bile duct cancer progression by activating WNT signaling [191]. These findings underscore the potential of combining PDO models with single-cell multi-omics technologies to explore tumor biology and drug sensitivity in BTCs [192]. However, a key challenge in current BTCs organoid research is how to integrate organoid models with gene editing and omics sequencing technologies to uncover the spatiotemporal regulatory changes during the early stages of BTCs, elucidate the molecular mechanisms of drug resistance, and define the molecular characteristics of the epithelial heterogeneous populations in BTCs.

#### The PDX model revealed drug sensitivity

Xenograft models, particularly cell line-derived xenograft (CDX) and patient-derived xenograft (PDX) models, are widely used in cancer research [193]. The CDX model is straightforward in establishing and producing consistent genetic and phenotypic traits, making it suitable for drug screening, evaluating drug combinations, and assessing the effects of treatments on tumor growth and survival. However, the CDX model has limitations, such as its tendency to become homogeneous during culture, which may not accurately represent the genomic diversity or the complex microenvironment of the primary tumor. In comparison, the PDX model preserves the genetic and phenotypic diversity of the patient's tumor, including the heterogeneous TME. This makes the PDX model an ideal tool for validating candidate drugs, identifying biomarkers for drug sensitivity or resistance, and evaluating the effects of treatments on metastasis and recurrence [194–197]. Hong et al. established a PDX model using patient-derived tumor tissues (such as KKU-368 and TJCHOL30) to replicate the heterogeneity of clinical tumors and evaluate the inhibitory effects of drugs (such as everolimus and IACS-010759) on specific CCA subtypes [81]. Zhan et al., focusing on GBC, developed a rapid mini-PDX model capable of completing chemotherapy drug sensitivity tests within 7 days. In this model, tumor cells from patients were implanted into subcutaneous capsules of nude mice to evaluate the efficacy of five drugs, including gemcitabine and oxaliplatin. The results showed that chemotherapy regimens based on PDX screening significantly extended the median overall survival (18.6 vs. 13.9 months) and disease-free survival (17.6 vs. 12.0 months) of patients, demonstrating the clinical value of PDX in guiding personalized treatment.

Furthermore, in conjunction with single-cell multi-omics analysis, the study revealed that drug sensitivity was closely linked to tumor heterogeneity. For instance, the effectiveness of irinotecan was associated with tumor size, lymph node metastasis, and TNM stage, while gemcitabine sensitivity correlated with nerve invasion. This suggests that the PDX model accelerates drug screening and facilitates the analysis of drug response variations among tumor subgroups by integrating molecular markers (such as p53 and P-gp), providing a foundation for targeted therapy in the complex TME [198]. Hernandez et al. further investigated the generation strategy of the PDX model, highlighting the feasibility of successfully creating PDX models from clinical biopsy samples of unresectable or metastatic BTCs, including those obtained through surgery or image-guided methods. The study found that the success rate for creating PDX models from metastatic lesions (69%) was significantly higher than that of primary unresectable tumors (15.4%) and that surgical biopsy samples (73%) were more effective for modeling than image-guided samples (14%). This discovery provides a valuable resource for studying the heterogeneity and metastatic mechanisms of advanced BTCs [199]. The PDX model, which closely mimics the tumor characteristics of patients, has become a critical link between basic research and clinical application [200, 201]. Its use in biliary tract tumors accelerates drug development. It offers an invaluable tool for studying tumor heterogeneity, the dynamic changes in the TME, and the development of personalized treatment strategies. Future research should focus on integrating single-cell transcriptomics, spatial omics, and immune microenvironment analysis to examine the interactions between tumor cells and the matrix in the PDX model, facilitating the development of novel combination therapies for the TME. Furthermore, expanding the sample size and including more biliary subtypes, such as iCCA, will increase the generalizability of the PDX model and drive the broader adoption of precision medicine from research to clinical practice(Fig. 4c).

### Improvement and future direction of single-cell multi-omics

Researchers should aim to shorten the ex vivo time by using fresh surgical or biopsy samples to minimize cell death. Bile duct epithelial cells can be enriched through tissue microdissection (LCM) or antibody labeling (such as EpCAM^+^). Developing mild dissociation enzyme combinations, such as collagenase IV + hyaluronidase, can help preserve cell activity and improve the quality of cell data. Following this, the epigenetic characteristics of the cells can be confirmed through multi-omics integration [202]. For example, Reduzzi et al. developed a novel liquid biopsy approach to address the limitations of traditional EpCAM-dependent circulating tumor cell (CTC) detection methods, such as CellSearch, in BTCs. They identified all CTC subsets through unbiased enrichment and single-cell analysis, including epithelial type (eCTC) and non-traditional type (ncCTC). They evaluated their potential applications in prognosis prediction, treatment monitoring, and molecular mechanism research [203].

Currently, there is a lack of widely adopted in situ implantation models derived from genetically engineered mice and patients in BTC research, limiting the ability of BTC mouse models to fully replicate the heterogeneity and immune microenvironment characteristics of human tumors. Although studies on PDO and PDX models have been conducted, they have not yet been fully utilized or promoted for BTCs. The development of a large-scale, high-throughput PDO/PDX model platform is eagerly awaited. Furthermore, the creation of in situ tumor models for BTCs and genetically engineered mouse models will be crucial for advancing animal models that more accurately simulate the origin and progression of human BTCs. The progression mechanisms of BTCs can be further analyzed using longitudinal single-cell sampling combined with trajectory inference algorithms, such as Monocle3 [204–207].

Single-cell technology alone cannot capture the spatial distribution of cells in situ. However, when combined with spatial transcriptomics (Visium) or multiplex fluorescence imaging (CODEX), it identifies interaction hotspots between malignant cells and immune/stromal cells. Using CODEX multichannel imaging, Baretti et al. performed high-parameter spatial immunophenotypic analysis on 24 iCCA samples. Their study uncovered the unique TME characteristics of FGFR2 and IDH1 mutant subtypes. Through single-cell level spatial omics analysis, this research clarified the immune microenvironment remodeling mechanism driven by molecular mutations, offering a foundation for designing immune combination therapies targeting specific molecular subtypes, such as FGFR inhibitors paired with immune checkpoint blockade or IDH1 inhibitors combined with immunotherapy [208]. Calderaro et al. integrated spatial transcriptome data with deep learning techniques to reclassify mixed hepatocellular carcinoma—cholangiocarcinoma (cHCC-CCA). Their findings confirmed a strong correlation between AI predictions, gene expression, mutation profiles, and patient prognosis [209]. Optimizing the technical process through interdisciplinary collaboration and combining single-cell data with functional experiments, such as organoid drug sensitivity tests, is essential. This approach will ultimately facilitate the clinical translation of BTC-targeted therapies, such as interventions targeting the CXCL12/CXCR4 axis.

## Conclusions

BTCs remain among the most challenging gastrointestinal malignancies due to their high heterogeneity, aggressive behavior, and limited treatment options. The advent of single-cell multi-omics technologies has profoundly transformed our understanding of BTC biology, enabling unprecedented resolution in dissecting tumor heterogeneity, microenvironmental remodeling, and clonal evolution.

Single-cell genomics, transcriptomics, epigenomics, and spatial transcriptomics and so on have collectively revealed the molecular diversity within and between BTC subtypes, identifying key driver mutations, immune evasion mechanisms, and stromal interactions that underlie disease progression and therapy resistance. These insights have not only refined existing molecular classifications but also uncovered novel therapeutic targets, such as immunosuppressive ligands, metabolic enzymes, and lineage-specific transcription factors. Moreover, the integration of multi-omics data with functional studies has highlighted the dynamic interplay between tumor cells and the TME, emphasizing the roles of TAMs, CAFs, Tregs, and exhausted T cells in fostering an immunosuppressive niche. These findings provide a strong rationale for combining targeted therapies with immunotherapy to overcome resistance and improve patient outcomes.

Despite these advances, several challenges remain. The application of single-cell technologies in BTC is still in its early stages, with limited sample sizes and a lack of multi-institutional cohorts. In the future, researchers should focus on developing highly sensitive, cost-effective spatial multi-omics technologies and integrating artificial intelligence algorithms (such as neural networks) to improve the analysis capabilities of multi-omics data and study the spatiotemporal dynamics of tumor cell interactions with the TME [210, 211]. Establishing a large, multi-center sample database, including BTC patients with varying etiologies and stages and long-term follow-up data, will help reveal the clinical significance of heterogeneous evolution. Using organoid models and CRISPR screening technologies, the regulatory networks of key driver genes can be validated at the functional level while exploring the impacts of metabolic reprogramming and epigenetic modifications on the TME to deepen our understanding of these mechanisms.

In conclusion, single-cell multi-omics represents a transformative approach for decoding the complexity of BTCs. By elucidating the molecular mechanisms driving tumor progression and treatment resistance, these technologies pave the way for precision medicine strategies tailored to individual patients’ tumor biology. Continued innovation in computational integration and experimental techniques will be essential to translate these insights into clinical practice and ultimately improve survival for patients with BTCs.

## Data Availability

All data and material are available in the main text.
